# Thrombosis of the right iliac, femoral, popliteal, and tibial arteries in a post-COVID-19 in adolescent

**DOI:** 10.1186/s43159-021-00124-5

**Published:** 2021-09-07

**Authors:** Mariana Orate Menezes da Silva, Henrique Amorim Santos, Amanda Fernandes Vidal da Silva, Guilherme Marum, Jose Maria Pereira de Godoy

**Affiliations:** 1 Service Vascular Surgery, Medicine School in Sao Jose do Rio Preto (FAMERP), São Jose do Rio Preto, Brazil; 2 Cardiology and Cardiovascular Surgery Department in Medicine School of Sao Jose do Rio Preto-FAMERP, São Jose do Rio Preto, Brazil; 3 CNPq (National Council for Research and Development), Rua Floriano Peixoto, São Jose do Rio Preto, SP 2950 Brazil

**Keywords:** Thrombosis, Iliac, Femoral, Popliteal, Tibial, Arteries, COVID-19, Adolescent

## Abstract

**Background:**

Viral infection into lung, muscular, and endothelial cells results in inflammatory response, including edema, degeneration, and necrotic alterations. The involvement of the major arteries in adolescent with COVID-19 has been infrequently reported in the literature. The aim of the present study is to report thrombosis of the right iliac, femoral and tibial arteries and stenosis of left iliac artery in an adolescent with COVID-19 and to discuss the pathophysiological hypotheses.

**Case presentation:**

We report the case of a 17-year-old female patient with COVID-19 infection. She was seen at the physician specialized general medicine in her hometown, was diagnosed with COVID-19 but did not require hospitalization. After 15 days, she had sudden pain in the left leg that has limited her ability to walk more than 10 met, associated with extremity cyanosis and coldness. Angiotomography revealed thrombosis of a portion of the iliac and popliteal arteries. Na emergency embolectomy was successfully performed, followed by full-dose heparinization with unfractionated heparin.

**Conclusion:**

Arterial thrombosis of large arteries may be associated with chronic inflammatory syndrome secondary to COVID-19 infection and the treatment with a late embolectomy was successful, even in a thrombotic event.

## Background

Viral infection into lung, muscular, and endothelial cells results in inflammatory response, including edema, degeneration, and necrotic alterations [1]. A study associates these lesions with the action of pro-inflammatory cytokines, including interleukin (IL) 6 and 10 and tumor necrosis factor α, granulocyte colony stimulating factor, monocyte chemoattractant protein-1, macrophage inflammatory protein 1α, increased expression of programmed cell death 1, T cellular immunoglobulin, and mucin domain 3 (Tim-3) [2].

Recent reports from Europe and the USA support the emergence of a new phenomenon with significant hyper inflammatory response in previously healthy asymptomatic children related to SARS-CoV-2 infection [3]. The term multisystem inflammatory syndrome (or MIS-C) has been given to this condition by the Centers for Disease Control and Prevention (CDC) [4]. Most reports describe significant gastrointestinal manifestations, such as vomiting, diarrhea, and severe abdominal pain.

A study evaluating which organic system were involved included gastrointestinal system in 171 patients (92%), cardiovascular in 149 (80%), hematological in 142 (76%), mucocutaneous in 137 (74%), and respiratory in 131 (70%) patients. Coronary artery aneurysms have been documented in 15 patients (8%), and aspects similar to Kawasaki’s disease have been documented in 74 (40%) patients [5]. The involvement of the major arteries in adolescent with COVID-19 is not very much reported in literature. The aim of the present study is to report thrombosis of the right iliac, femoral and tibial arteries, and stenosis of left iliac artery in an adolescent with COVID-19 and to discuss the pathophysiological hypotheses.

## Case presentation

We report the case of a 17-year-old female patient with COVID-19 infection. She had fever, nausea, diarrhea, headache, myalgia, and mild dyspnea*.* She was seen at the physician specialized general medicine in her hometown and was diagnosed with COVID-19 but did not require hospitalization. This patient was medicated in her hometown with *loratadine* and *azithromycin*. The most intense clinical features lasted 7 days and then were mild for a week. After 15 days, she had sudden pain in the left leg that has limited her ability to walk more than 10 m, associated with extremity cyanosis and coldness, but she sought medical care after a week and was referred to a specialized service in a quaternary teaching Hospital.

**Fig. 1:**
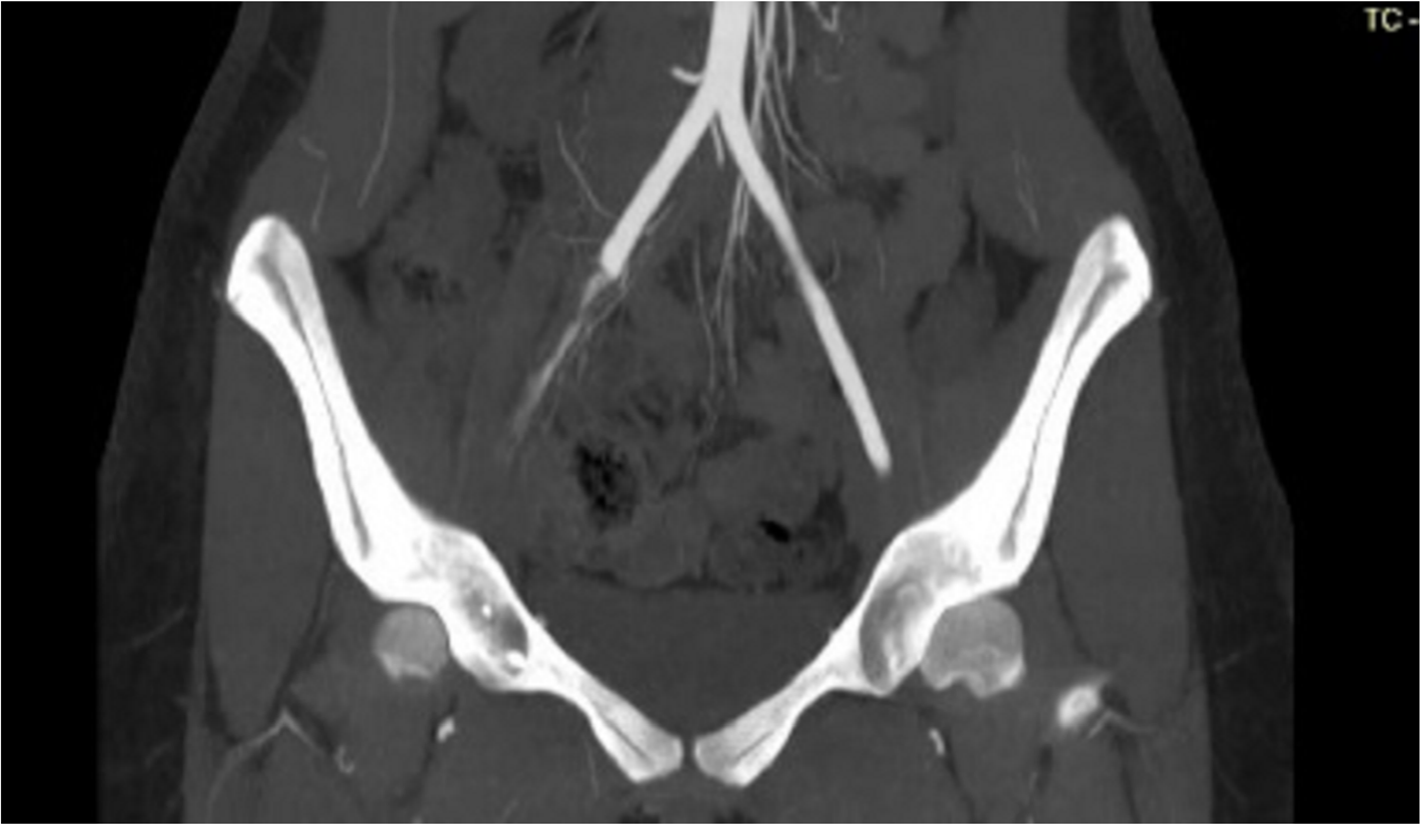
shows the iliac artery thrombosis

**Fig. 2:**
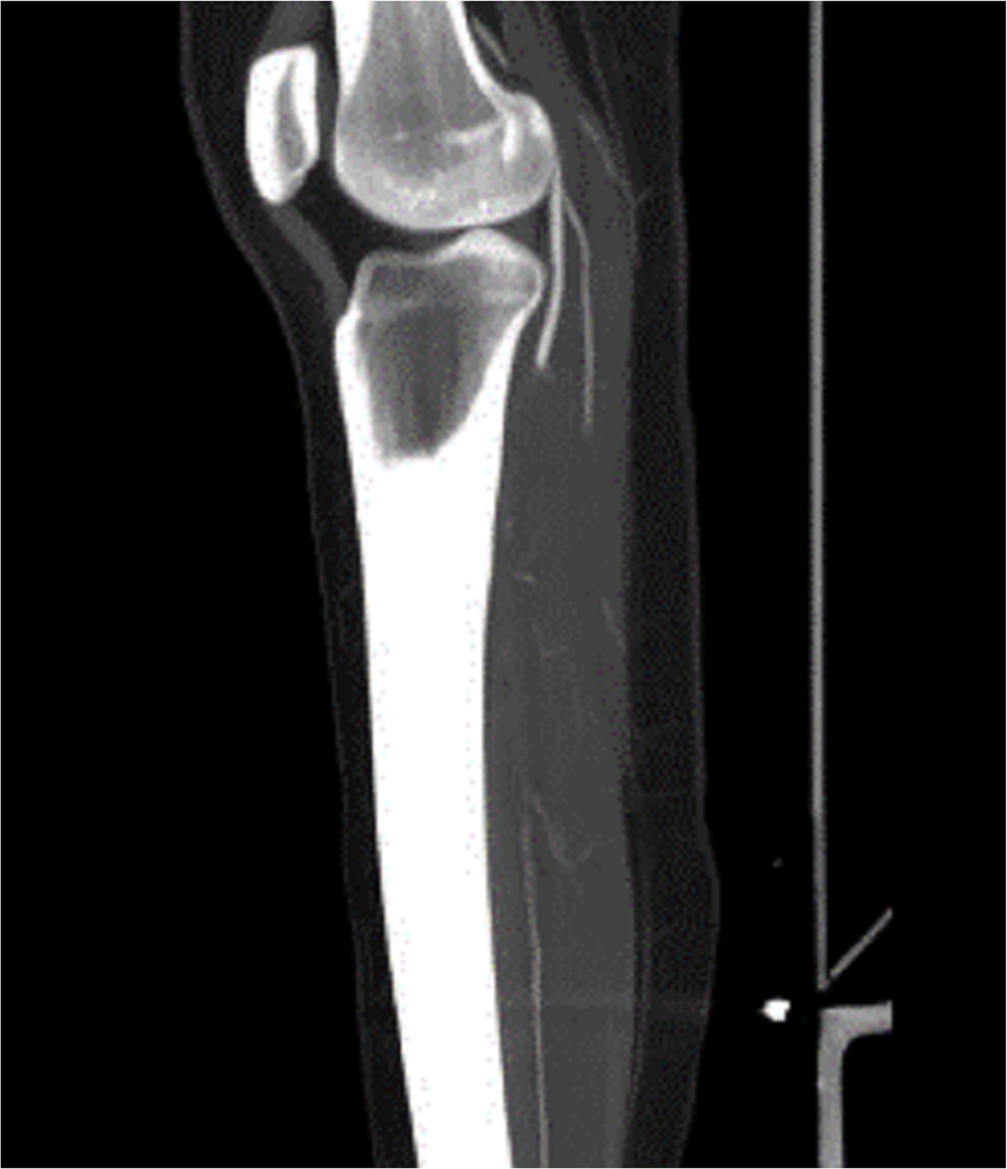
shows thrombosis of the popliteal artery

A clinical history and physical examination were performed, in which the absence of all pulses (femoral, popliteal, anterior, and posterior tibial arteries) of the left leg was detected, with coldness and mild cyanosis, which brought the diagnosis hypotheses of acute arterial ischemia. It was confirmed by computerized angiotomography (angio-CT) that revealed thrombosis of a portion of the iliac and popliteal arteries, as shown in Figs. 1 and 2. In the complementary exams, the platelet count was 83,000, and the reactive protein-C was elevated and the oxygen saturation was less than 93%. Na emergency embolectomy was successfully performed, followed by full-dose heparinization with unfractionated heparin. The arterial thrombotic condition responded well to the treatment and had an ankle/arm index of 1. Chest CT showed moderate pericardial effusion, absence of intracardiac thrombi, and an enlarged cardiac area. Bilateral pleural effusion, however, does not have signs of thrombosis or pulmonary thromboembolism, with the presence of ground-glass images in the peri-bronchial and basal regions. The study was approved Committee Ethical Research in Medicine School of Sao Jose do Rio Preto# 4.300.416. The participant signed an informed consent form.

## Discussion

The present study reported a case of acute arterial thrombosis of multiple arterial segments of the right iliac, femoral, popliteal, and tibial arteries associated with COVID-19 in adolescent. The patient had a non-severe initial clinical manifestation of COVID-19 infection which did not require hospitalization, with mild respiratory symptoms and mainly gastrointestinal complains. The patient had a late symptoms onset of acute arterial ischemia with limitation to walk and intense pain. There was a delay of a week before being referred to a specialized center.

Embolectomy was the chosen therapy, despite the long duration of ischemia, but it occurred without complications and with reperfusion of the limb. Embolectomy is not the first option in cases of arterial thrombosis, where the bypass or endovascular procedure is recommended. The cause of the arterial thrombotic event, of a large artery, is the absence of available previous publications in children. Therefore, the main hypothesis of this thrombotic event was the COVID-19 infection. Inflammation of the vascular system can result in diffuse micro angiopathic thrombi, inflammation of the heart muscle (myocarditis) and cardiac arrhythmias, heart failure, and acute coronary syndrome [6].

Activated macrophages can release cytokines, including IL-1β and IL-6, which will promote the expression of adhesion molecules for endothelial activation, inflammatory cell infiltration and vascular inflammation. Endothelial cells release pro-inflammatory cytokines that contribute to the spread of microcirculatory lesions [7]. A clinically significant effect of inflammation is coagulopathy and the dysfunctional endothelium becomes pre-adhesive and procoagulant leading to an auto-index of venous thromboembolism [8, 9].

The literature data focus more on adults, but children tend to have milder clinical conditions compared to adults. However, thrombosis of the great arteries is uncommon in adults and very rare in children. Chronic inflammatory syndrome associated with Kawasaki disease has been reported as a probable cause in children, but the clinical presentation is usually more severe.

The endothelial lesion associated with hypercoagulability, resulting from the chronic inflammatory process, is the most acceptable hypothesis in the present study. The fact that the child spent a week with reduced symptoms reinforces the hypothesis that a chronic inflammatory syndrome can cause serious complications even after the most symptomatic period of the disease. Congenital and acquired thrombophilia are more frequently associated with venous/non-arterial clinical conditions. Therefore, reinforcing the hypothesis of hypercoagulability and inflammatory changes associated with COVID-19 infection.

Arterial thrombosis of large arteries may be associated with chronic inflammatory syndrome secondary to COVID-19 infection and the treatment with a late embolectomy was successful, even in a thrombotic event.

## Conclusions

Arterial thrombosis of large arteries may be associated with chronic inflammatory syndrome secondary to COVID-19 infection and the treatment with a late embolectomy was successful.

## Data Availability

All data are available in the text.

## Abbreviations

SARS-COV2: Respiratory syndrome coronavirus-2
MISC-C: Multisystem inflammatory syndrome
CDC: Disease Control and Preventive
CT: Computed tomography
COVID-19: Coronavirus disease 2019
Angio-CT: Angiotomography
